# Malachite Green Dye Decoloration over Au/TiO_2_-Nanotubes Photocatalyst under Simulate Visible-Light Irradiation

**DOI:** 10.3390/ma15186209

**Published:** 2022-09-07

**Authors:** María Guadalupe Hernández-Cruz, Dora Alicia Solís-Casados, José Antonio Toledo-Antonio, Jorge Roberto Vargas-García, Miriam Estrada-Flores, Carlos Ángeles-Chávez, María Antonia Cortés-Jácome, Cecilia Encarnación-Gómez

**Affiliations:** 1 División Académica Multidisciplinaria de Jalpa de Méndez, Universidad Juárez Autónoma de Tabasco, Carr. Villahermosa-Comalcalco Km 27 S/N, Ranchería Ribera Alta, Jalpa de Méndez 86205, Mexico; 2 Centro Conjunto de Investigación en Química Sustentable UAEM-UNAM, Universidad Autónoma del Estado de México, Toluca 50200, Mexico; 3 Instituto Mexicano del Petróleo, Eje Central Lázaro Cárdenas # 152, Ciudad de México 07730, Mexico; 4 Instituto Politécnico Nacional, Depto. De Ing. Metalúrgica, Ciudad de México 07300, Mexico; 5 Instituto Politécnico Nacional, Escuela Superior de Ingeniería Química e Industrias Extractivas, DIQI, Ciudad de México 07330, Mexico

**Keywords:** titania nanotubes, photocatalytic decoloration, vapor-phase impregnation, gold nanoparticles

## Abstract

Au nanoparticles were supported on TiO_2_ nanotubes by a novel vapor phase impregnation approach (VPI) using gold dimethyl-acetylacetonate as a precursor. This study aimed to evaluate the capacity of these materials in the photodecoloration of malachite green dye, with the vision to correlate the chemical, structural, morphological, and optical properties with its photocatalytic performance. The photocatalysts were characterized by X-ray diffraction, Raman spectroscopy, X-ray photoelectronic spectroscopy (XPS), electronic microscopy (HAADF-STEM and HRTEM), and UV–vis spectroscopy. The techniques mentioned above made it possible to detect the presence of small gold nanoparticles (around 3.1 nm), with a high apparent dispersion even at high metal loading for all analyzed systems. According to the XPS results, the Au nanoparticles remain reduced (Au°), and they have a high electronic interaction with TiO_2_, which eventually originates an electronic exchange between them and consequently a decrease in the band gap energy. In addition, the surface plasmonic resonance observed through UV–vis spectroscopy of the Au nanoparticles are factors that can be related to the high decoloration observed in these photocatalysts, specifically in the 15 wt% Au material, which achieves maximum photodecoloration of malachite green dye at 93%.

## 1. Introduction

Malachite green (MG) is regarded as one of the most toxic and persistent dyes using commonly in the textile, food, and aquaculture industries that ends up being part of wastewater [1,2,3,4]. For this reason, MG dye has been used as a model molecule to evaluate the capacity of decoloration of catalytic materials with different chemical natures, crystalline structures, morphology, and other critical physicochemical properties that can impact its photocatalytic performance [1,5,6]. In this sense, the photocatalytic process emerged as a feasible alternative among the advanced oxidation processes since it allows it to work at room temperature and under UV or solar light sources. Some materials have photocatalytic properties, including metal-transition oxides, such as TiO_2_, CeO_2_, and WO_3_, and include other semiconductors [7,8,9]. Nevertheless, the challenge remains to find materials that increase photocatalytic efficiency through more affordable energy sources (visible light) and contribute to understanding the relationship between the properties and their photocatalytic performance in some chemical reactions of environmental interest and many other applications [10,11]. 

In this quest for innovation in improved materials, nanostructured titanium oxide has attracted considerable attention because it can be used as a catalytic material or support for other active phases [3,12,13,14,15]. Particularly, TiO_2_ nanotubes (TiO_2_-NT), due to their textural properties and functional groups, promote an increase in the number of reaction sites and their dispersion [16]. The use of TiO_2_ nanotubes was widely reported in hydrotreatment reactions. In them, the high surface area of this material (~350 m^2^/g) has allowed obtaining a higher catalytic activity in comparison with other TiO_2_-based supports [17,18,19]. Additionally, in photocatalytic and photoelectrochemical reactions, TiO_2_ nanotubes present a high degree of control over the separation of photogenerated charge carriers, improving the efficiency of the processes [20,21]. A strategy already studied to reduce the band gap of TiO_2_ consists in the addition of noble metals, which can modify its electronic state and avoid the recombination process of the electron-hole pair, and at the same time, it is possible to extend the absorption of light to the visible range [22,23,24,25]. 

Specifically, nanostructured metals such as gold, silver, and copper exhibit resonant behavior when interacting with ultraviolet and visible photons, so solar energy can be mostly used in various photoinduced chemical reactions [26]. In this sense, the coupling between plasmonic metals and semiconductors has enabled it to obtain photocatalysts that reach higher reaction rates than their pure semiconductor counterparts [26,27,28]. In particular, the Au/TiO2-NT system shows a synergistic effect, in which TiO2-NT promotes photoinduced charge separation and reduces the recombination process by delocalizing charge carriers. Meanwhile, gold nanoparticles act as an electron trap to reduce electron-hole recombination, and its surface plasmon resonance promotes a wide range of absorption in the visible light region [29,30]. In this respect, Au nanoparticles on TiO_2_-NT material were tested as photocatalysts in degradation reactions [30,31], antibacterial and anti-inflammatory agents [32], photoelectrochemical biosensors, and photosensors [25,33]. Few studies reported the use of TiO_2_ nanotubes for malachite green photodegradation, and to our knowledge, none of these consider TiO_2_ nanotubes with Au nanoparticles despite the novel electronic properties shown by this system. For example, a comparative study of photocatalytic performance between TiO_2_ anatase nanoparticles and TiO_2_ nanotubes demonstrated that the nanotubes completed the degradation reaction in a shorter time than TiO_2_ anatase nanoparticles [16]. 

Like other catalytic systems, the dispersion, particle size, and surface area of active phases constituted important issues that impact photocatalytic performance. In turn, the manipulation of the composition, shape, and size allows the generation of plasmonic particles that interact with sunlight more effectively [34]. For this purpose, the metal incorporation on nanotubular support can be carried out by several methods of synthesis such as reduction reaction in aqueous solution [30], electrochemical deposition [33,35], magnetron sputtering [36,37], sol–gel [38], deposition precipitation with urea [39] and wet impregnation [40]. Equally, through the vapor-phase methods, as vapor-phase impregnation, it is possible to obtain high dispersion of metal particles on different kinds of supports, especially those with surface functional groups that can serve as anchorage sites and, in turn, inhibit particle growth [41]. In this context, the present contribution reports the photocatalytic evaluation of Au nanoparticles supported on TiO_2_ nanotubes synthesized by vapor phase impregnation methodology. This method of particle incorporation on nanostructured TiO_2_ support allowed us to achieve an acceptable dispersion of the active phase in the reduced state with controlled particle size independent of the metal loading. The correlation between the physicochemical properties and the photocatalytic performance of these materials was discussed.

## 2. Materials and Methods

### Materials Synthesis

**Figure 1:**
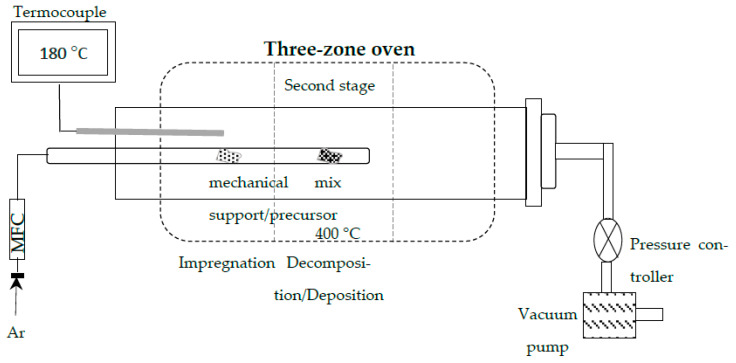
Schematic representation of vapor phase impregnation methodology.

Titania nanotubes were prepared by hydrothermal method using a NaOH solution of 10 M in an autoclave reactor, maintaining autogenous pressure under experimental conditions previously reported [42]. The Au nanoparticles incorporation on TiO_2_ nanotubes was carried out by the vapor-phase impregnation (VPI) method, using a horizontal furnace. A mechanical mixture in agate mortar between powder precursor and support was developed before introducing the reaction system. The nominal gold content deposited on TiO_2_-NT was 1, 3, 6, and 15 wt%. The two-step approach includes sequential stages, where the mixed powder was heated at 180 °C to reach the sublimation of Au precursor [(CH_3_)_2_(C_5_H_7_O_2_) Au–98%], immediately followed by the second step at 400 °C to achieve the gold precursor decomposition and to deposit the Au particles on TiO_2_ nanotubular surface. A stream of 100 sccm of Ar carrier gas was supplied to favor the interaction between the metal precursor and the support interaction, as well as the evacuation of secondary reaction gases. A total pressure of 500 Torr was maintained in the reactor system. Figure 1 shows a schematic representation of the vapor phase impregnation methodology for depositing gold particles on TiO_2_ nanotubes. The characterization and photocatalytic evaluation of Au/TiO_2_ nanotube materials were performed without prior treatment. 

## 3. Materials Characterization

### 3.1. X-ray Diffraction

A Bruker D8 Advance X-ray Diffractometer using monochromated Kα Cu radiation (X-ray source 2.2 kW, running conditions were 40 kV and 40 mA) was used to determine the structural characteristics of Au/TiO_2_-NT materials. Measurements were performed in the 2θ range from 10° to 90° with a step of 0.02°.

### 3.2. Raman Spectroscopy

Raman spectra of the samples were recorded with a Perkin Elmer spectrophotometer model Spectrum GX in a spectral range of 100 to 2500 cm^−1^ with a 1064 nm laser.

### 3.3. HAADF-STEM and HRTEM

The morphological characteristics of Au-TiO_2_-NT material were carried out by Transmission electron microscopy (TEM) and scanning transmission electron microscopy (STEM). Both techniques were conducted in a JEM-2200FS–Jeol microscope with an accelerating voltage of 200 kV. The microscope is equipped with a Schottky-type field emission gun, and an ultra-high-resolution configuration (Cs = 0.5 mm; Cc = 1.1 mm; point-to-point resolution = 0.19 nm) and in-column omega-type energy filter. The microscope operates with an aberration-corrected device CEOS in STEM mode, producing a significantly smaller and brighter electrons beam, a probe size of 0.1 nm. High angle annular dark field (HAADF) image was obtained using the HAADF detector in the STEM mode. In this technique, the detector collects electrons that undergo Rutherford scattering, where their intensities are approximately proportional to Z2 (Z being the atomic number of the scattering atom). Elements with a high Z show higher intensities and brighter contrast in the image. In the images, the difference in contrast between the support and the metal particles made it possible to determine the particle size of each specimen through the measurement of about 200 particles. The samples were ground, suspended in isopropanol at room temperature, and dispersed with ultrasonic agitation. An aliquot of this solution was then dropped onto a 3 mm in diameter lacey carbon copper grid.

### 3.4. X-ray Photoelectronic Spectroscopy (XPS)

The Au oxidation state and the surface chemical composition were determined by XPS. The XPS spectrum was obtained on a Thermo-VG Scalab 250 spectrometer equipped with an Al Kα–X-Ray source (1486.6 eV) and a hemispheric analyzer. 

The experimental peaks were decomposed into individual components using mixed Gaussian–Lorentzian functions, non-linear squares fitting algorithm, and Shirley-type background subtraction by using XPS peak fit software. The C 1s line at 284.6 eV was used as an internal standard for correcting binding energies (BE).

### 3.5. UV–Vis Spectroscopy

The UV–Vis spectroscopic technique was used to obtain the absorbance spectra of Au-TiO_2_-NT materials to obtain the band gap energy and track the progress of the decoloration reaction. For this purpose, we use a UV–Vis spectrophotometer (Perkin Elmer LAMBDA 35). The measurement wavelength range was from 200 to 1000 nm. The optical properties of solid materials were determined using an integration sphere and Spectralon as a reference; this material has a reflectance generally >95% from 250 to 2500 nm. 

### 3.6. Photocatalytic Evaluation

The decoloration capacity under solar light of Au/TiO_2-_NT materials was evaluated using a system constituted by a light source emitted via a solar simulator Scientech SF150 model, class A, with an average intensity of 60 mW cm^−2^. The distance between the light source and the reaction system was 15 cm. The photocatalytic reaction was carried out using 25 mL of malachite green carbinol base dye solution 10 μmol L^−1^. All experiments were carried out at room temperature. The dye decoloration was followed by taking aliquots of 4 mL at different times through absorbance measurement (616 nm malachite green) and related with a concentration using the Lambert–Beer equation [43]. The kinetic study of malachite green decoloration was developed considering a pseudo first-order kinetic order from the reaction time vs. concentration graph. Before the dye decoloration reaction, the photocatalysts were placed in the malachite green solution without light irradiation for a stabilization time of 30 min.

## 4. Results

### 4.1. X-ray Diffraction

**Figure 2:**
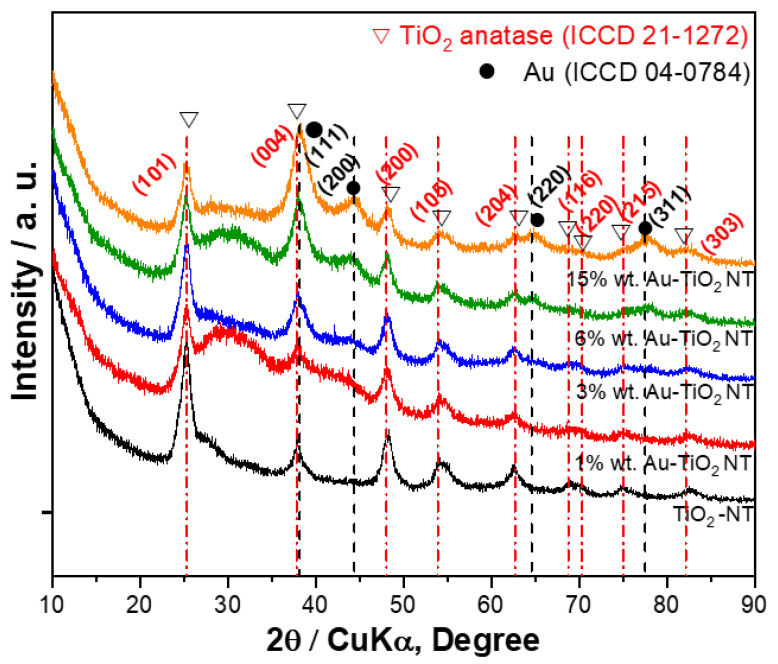
X-ray diffraction patterns of Au/TiO_2_-NT photocatalysts with different metal loads.

The structural characteristics of Au/TiO_2_-NT were investigated by the X-ray diffraction technique. The X-ray patterns show the characteristic reflections attributed to the TiO_2_ anatase phase in all samples (ICCD 21-1272; Figure 2). Additionally, other reflections can be observed in materials with 3, 6, and 15 wt% Au at 2θ = 44.3° and 64.7°, related to the FCC phase of Au (ICCD 04-0784). The most intense reflection (111) of Au at 38.1° is very close to (004) of the anatase phase at 37.8°. Consequently, both peaks can overlap; despite this, a significant increase in the intensity of the peak was observed. It is also notable that the (101) intensity decreases due to inhibition of the anatase phase with the gold incorporation. No other reflections attributed to any gold-related phases are appreciated in the diffraction patterns. It should be noted that only with Au contents of 6% or more the characteristic metal signal is appreciable, which suggests that the particles are very dispersed on the support. This is relevant considering that the gold nanoparticles are active sites that can interact with the dye molecule.

### 4.2. Raman Spectroscopy

**Figure 3:**
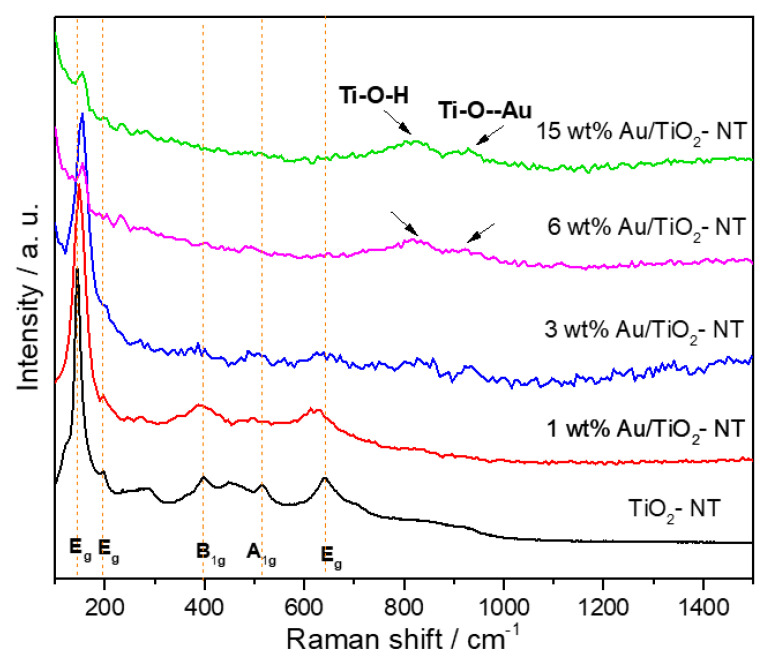
Raman spectra of Au/TiO_2_-NT photocatalysts with different metal loads.

The Raman spectra of the Au/TiO_2-_NT samples are shown in Figure 3. In addition, the spectrum of TiO_2_ nanotubes is shown. It can be seen the presence of vibration bands at 146, 196, 398, 515, and 640 cm^−1^, corresponding to E_g_ (1), E_g_ (2), B_1g_, A_1g_, and E_g_ (3), attributed to anatase phase of TiO_2_ [44]. Increment in the metal loading causes a significant decrease in the vibration modes of anatase, just like a slight change in the Raman shift. This is attributed to different factors, such as lattice defects and oxygen deficiencies, which can contribute to changes in the position, width, and shape of the Eg mode [45]. It is noteworthy that in the Raman spectra of the samples with 6 and 15 wt% Au, two broad vibration bands can be appreciated at 924 and 820 cm^−1^, approximately. These bands could be assigned to Ti–O–Au (not a formal bond) and Ti–O–H symmetrical stretching modes with very short Ti–O distance in titanate structures. The 905–920 cm^−1^ band suggests an incomplete ion exchange of Au, which is typical in the nanotubular titanate systems [46,47,48,49,50]. This can favor the charge transfer process between the metal and the semiconductor when it is photoactivated.

### 4.3. Scanning Transmission Electron Microscopy (STEM)

**Figure 4:**
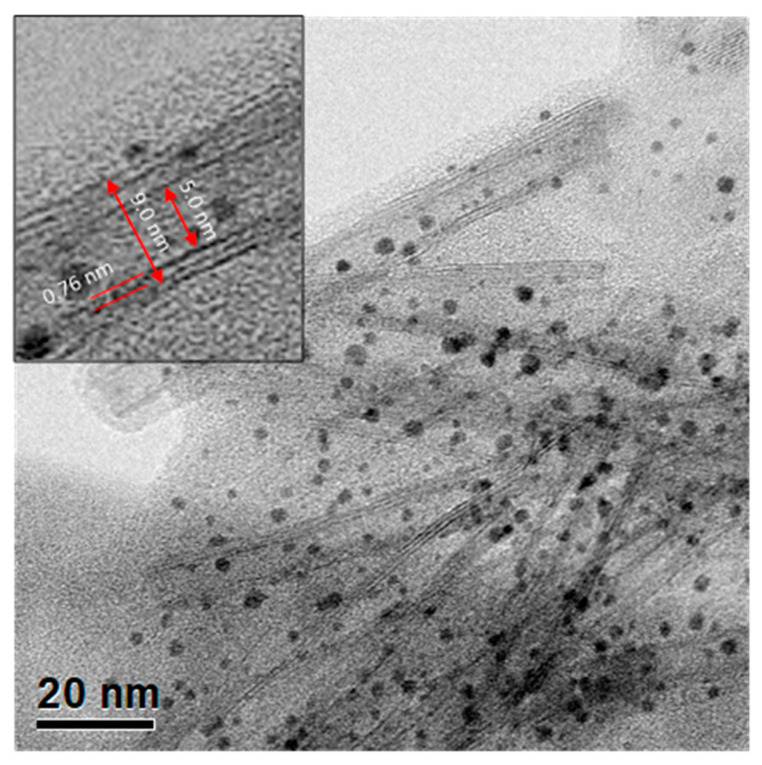
STEM image of TiO_2_ nanotubes decorated with Au nanoparticles (15 wt% Au photocatalyst).

**Figure 5:**
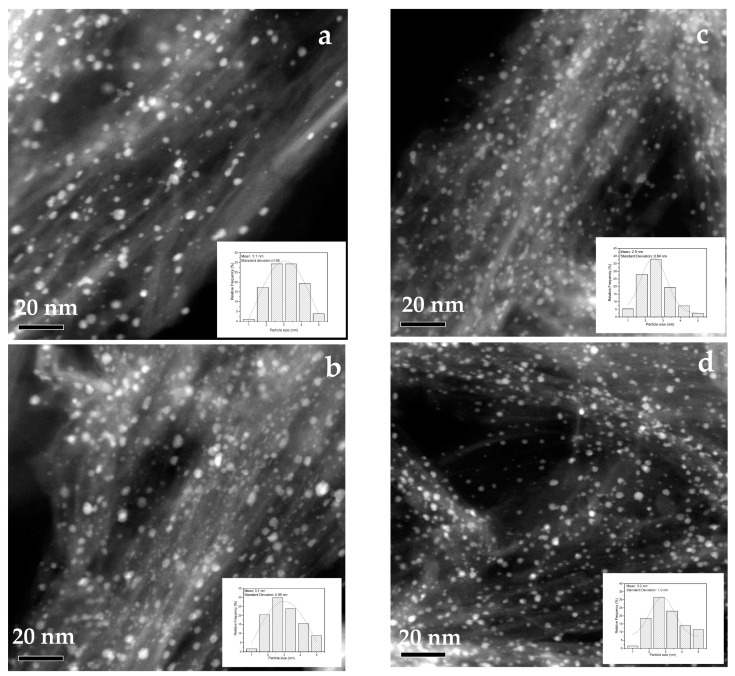
HAADF-STEM images of Au/TiO_2_-NT photocatalysts with different metal content and its corresponding histogram of particle size distribution. (**a**) 1 wt% Au, (**b**) 3 wt% Au, (**c**) 6 wt% Au, and (**d**) 15 wt% Au.

The surface morphology of TiO_2_ nanotubes decorated with gold nanoparticles (15 wt% Au loading) was observed by STEM and is presented in Figure 4. The nanotubes are composed of two structural layers with an interlayer distance of 0.76 nm; the inner diameter of the nanotubes is around 5.0 nm, whereas the outer diameter is around 9.0 nm (inset of Figure 4). The morphological study of TiO_2_ nanotubes prepared by the hydrothermal method was previously reported [51,52]. Uniform distribution of particles (black dots) with a relatively narrow range on TiO_2_-NT support was observed. Nevertheless, to determine the average particle size and confirm the chemical nature of nanoparticles more precisely, the samples were analyzed through STEM in the dark field mode (Figure 5).

### 4.4. HAADF-STEM

Figure 5a–d compares HAADF-STEM images for 1, 3, 6, and 15 wt% Au/TiO_2_-NT. The predominant feature of the samples is the high dispersion of bright spots with an apparent spherical geometry on the nanotubular TiO_2_ support, attributed to Au nanoparticles, given the difference in contrast of the images. The increase in the metal load up to 15 wt% Au seems not to induce the agglomeration of the particles, so the size of the particles remains almost unchanged at around 3.1 nm. Nevertheless, the standard deviation denotes a high data dispersion around the mean, indicating that Au nanoparticles prepared by vapor phase impregnation have a wide variety of sizes between 1 and 5 nm. The statistical analysis was performed by measuring 200 particles shown in the histogram inset of Figure 5a–d. In all samples, the mean size of Au nanoparticles remains constant at around 3 nm.

### 4.5. HR-TEM 

**Figure 6:**
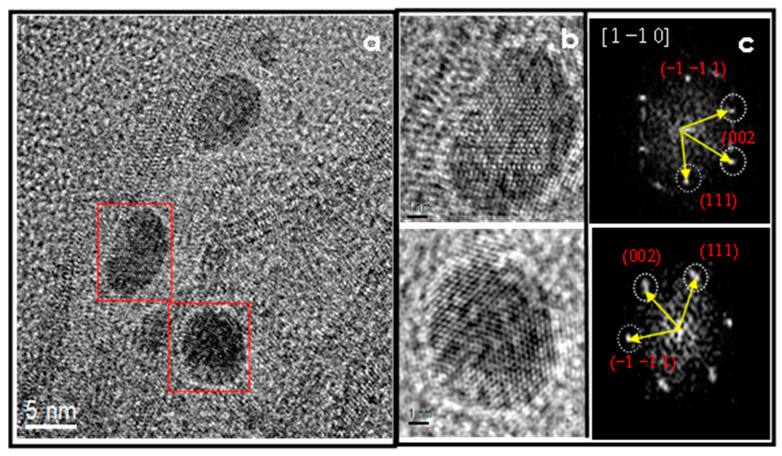
(**a**) HR-TEM image displaying crystalline nanoparticles decorating the TiO_2_ nanotubular support. (**b**) Magnified images of nanoparticles. (**c**) Fourier Transform image.

The complementary study of the morphology and crystalline structure of the Au nanoparticles on TiO_2_-NT was performed by HR-TEM. In the image of Figure 6a, can be observed small semispherical particles with a crystalline arrangement of about 5 nm in size. Two particles are enclosed in the red square, and its corresponding magnified image and Fourier Transformed are displayed in Figure 6 b and c. d—spacing of 0.232 nm, 0.231 nm, and 0.208 nm presents a good match with the (1 1 1), (−1 −1 1), and (0 0 2) planes, viewed along [1 −1 0] zone axis, attributed to the FCC crystal structure of Au, according to the ICCD card number 4-0784. Between the geometries associated with gold nanoparticle growth are classified as icosahedral and fcc polyhedral. Gold nanoparticles, prepared by vapor-phase impregnation, apparently crystallize in cuboctahedra or truncated cuboctahedra geometries.

### 4.6. X-ray Photoelectronic Spectroscopy

**Figure 7:**
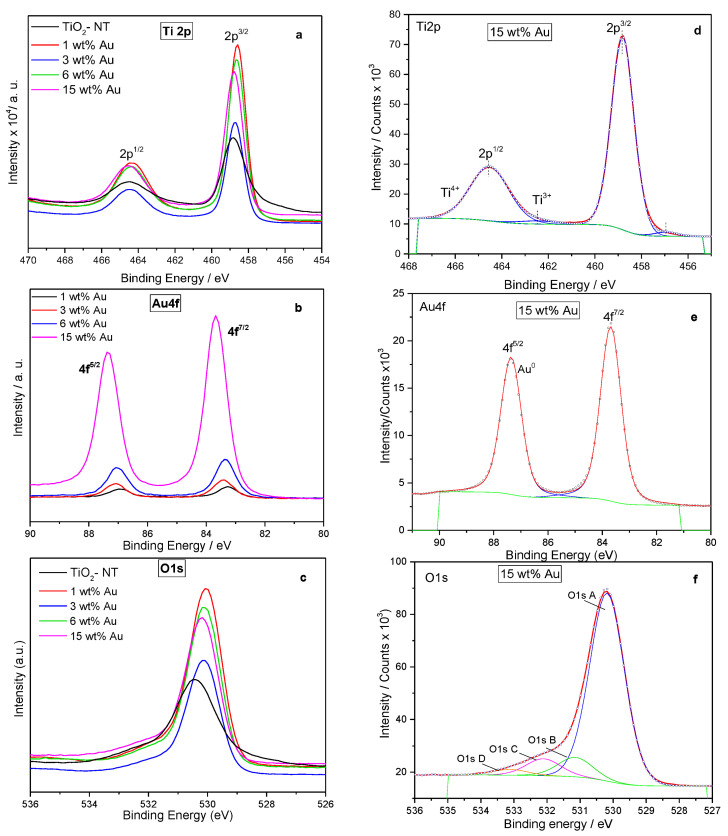
XPS spectra of Au/TiO_2_-NT photocatalyst with different metal content. (**a**–**c**). Comparison of XPS spectrum for Ti2p, Au4f, and O1s levels, respectively. (**d**–**f**). Deconvoluted Ti2p, Au4f, and O1s level graphs for 15 wt% Au.

The analysis of the chemical state of Au/TiO_2_-NT photocatalysts surface was carried out by High-resolution X-ray photoelectronic spectroscopy- XPS. Figure 7a–c shows the Ti2p, Au4f, and O1s XPS spectra of unmodified TiO_2_-NT, as well as 1, 3, 6, and 15 wt% Au/TiO_2_-NT. The comparison between spectra with different metal content allows observing a slight variation in the binding energy and intensity at energy levels analyzed with respect to the XPS spectra of the TiO_2_ nanotubes. For the TiO_2_-NT sample, it was possible to note that two pairs of peaks constitute the Ti2p region; the first one corresponds to the doublet Ti2p^1/2^ (binding energy, BE, 464.4 eV) and Ti2p^3/2^ (BE 458.8 eV) arises from spin orbit-splitting. The binding energy between Ti 2p^1/2^ and Ti 2p^3/2^ (5.8 eV) indicated that Ti exists in the form of Ti^4+^ in the TiO_2_ lattice [53,54]. The other detectable signals appear at lower binding energies (Ti2p^1/2^-463.4 eV and Ti2p^3/2^-457.5 eV), indicating the presence of a small contribution of reduced species Ti^3+^. The amount of reduced Ti^3+^ seems to decrease as Au content increases since Au is the component easier to reduce than Ti.

Figure 7d shows the deconvolution of the 15 wt% Au/TiO_2_-NT spectrum, where the same signals attributed to the presence of Ti^4+^ and Ti^3+^ ions can be observed, with slight variations in the binding energy due to the shift in the chemical environment of the TiO_2_ derived from the inclusion of Au [55]. Previous studies reported that the incorporation of metal ions in the support structure could originate the reduction in TiO_2_, either as a thin film or powder constituted by nanotubular geometry [56]. In some cases, the formation of Ti^3+^ ion reveals the presence of oxides such as Ti_2_O_3_ or some mixed oxide structure with de metal dopant [53,57], in this case, with gold, which is unlikely due to its chemical nature, so it is not considered as a formal bond. Even X-ray diffraction does not show the presence of other phases with gold. 

Likewise, the XPS spectra in the Au4f region show a significant increment in the intensity of the signals for 15 wt% Au photocatalyst (see the deconvoluted spectrum of 15 wt% Au-Figure 7e). In all the samples, the deconvoluted spectra with Au4f5/2 and Au4f7/2 in energy values around 87.4 and 83.7 eV, respectively, correspond to metallic gold. It is interesting that even at the highest Au concentration, all Au nanoparticles deposited on TiO_2_-NT remain in a reduced state as Au^0^, which can be reached by the vapor phase impregnation method used to prepare the samples. Additionally, the analysis of O1s core-level spectra is shown in Figure 7c. The deconvoluted spectrum of 15 wt% photocatalysts (Figure 7f) shows the superposition of four components centered at 529.8 (O1s A), 530.8 (O1s B), 531.7 eV (O1s C), and 532.6 (O1s D). O1s A peak is characteristic of O_2_^−^ in the TiO_2_ lattice; O1s B peak corresponds to O_2_- associated with the vacancy sites [58,59].

The higher binding energy O1s C band is related to some surface hydroxyl (OH) group, whereas the fourth peak at 532.6 eV corresponds to some residual H_2_O and/or carbonates generated during Au precursor decomposition [31]. The results described above allow us to deduce that due to the synthesis conditions of the photocatalysts, the reductive atmosphere generated during Au precursor decomposition reduces the Au nanoparticles, allowing us to obtain gold nanoparticles in a reduced state (Au°). The XPS parameters of Au/TiO_2_-NT photocatalysts are shown in Table 1.

### 4.7. UV–Vis Spectroscopy

**Figure 8:**
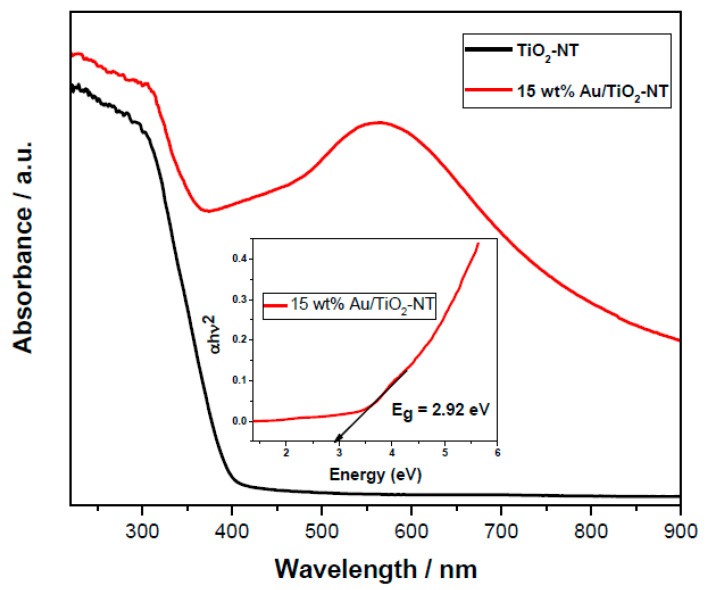
The UV–vis light absorption spectra of TiO_2_-NT and Au/TiO_2_-NT photocatalysts.

The optical properties of the synthesized TiO2-NT were investigated by UV–Vis spectroscopy. Figure 8 shows the corresponding spectra of pure TiO_2_ nanotubes and 15 wt% Au/TiO_2_-NT. The spectrum displays an intense absorption band between 200 and 400 nm, which is typical of semiconductor titania nanotubes [60]. In addition, the samples Au NP-containing presented an absorption component between 500 and 700 nm; however, only the spectrum with 15 wt% Au is shown in Figure 8; the spectra of the remaining photocatalysts show a similar trend with an increase in the absorption peak associated with the metal content. This band is attributed to the surface plasmonic resonance of Au nanoparticles (SPR) [28,61,62]. Photocatalysts constituted by semiconductors and plasmonic metal nanostructures, such as Au, interact with light through the excitation of SPR. Several studies showed that the SPR plays an important role in enhancing the rate of numerous photocatalytic reactions [63,64,65,66]. Therefore, Au-SPR increases the photocatalytic reaction rate by increasing the steady-state concentration of charge carriers at the surface of the semiconductor [34]. 

Moreover, it is known that TiO_2_-based photocatalysts exhibit band gap energies around 3.2 eV, and the incorporation of metal nanoparticles on TiO_2_-NT can change the band gap to the desired level. One way to evidence the possible changes in the electronic properties of titanium nanotubes is through the measurement of band gap energy. In the inset of Figure 8 can be appreciated the band gap determination via Kubelka–Munk function and related to the wavelength in the Tauc graph for 15 wt% Au photocatalyst. The results indicate that, indeed, the deposition of Au nanoparticles on TiO_2_-NT promotes a decrease in the energy values, and it is possible to associate it with an ion exchange process mentioned in the XPS analysis [49,67]. Furthermore, such a decrease in a band gap energy suggests a strong electronic interaction between nanotubular TiO_2_ and Au. Table 2 shows the results of the determination of band gap energy for all photocatalysts. 

### 4.8. Photocatalytic Evaluation

**Figure 9:**
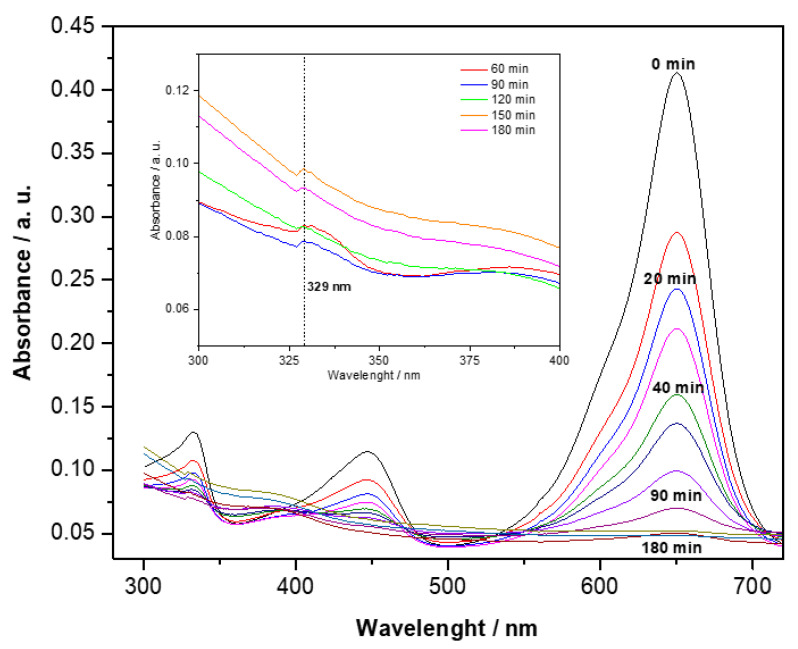
Absorption spectra of MG dye solution with 15 wt% Au/TiO_2_-NT photocatalyst at different reaction times.

The effect of Au content on the decoloration of malachite green dye was studied by UV–Vis spectroscopy through a reduction in the absorption bands characteristic of MG at 316, 425, and 616 nm [68]. Figure 9 shows the UV–vis spectra of the system photocatalyzed by 15 wt% Au/TiO_2_-NT sample at different reaction times. The monitoring of the most intense absorption band (616 nm) allowed us to observe the variation in the absorbance intensity as the reaction progressed. It is evident that as the irradiation time increases, the initial blue color of the solution gradually turns light-colored, and the intensity absorption band decreases (this appreciation will be observed later). The decrease in the absorption band intensity was attributed to the destruction of the whole conjugated chromophore structure of the dye [3]. In addition, a small band around 329 nm was observed, specifically after one hour of starting the reaction (inset of Figure 9 corresponds to the amplified region of the wavelength), which can be attributed to cleavage of the central carbon and possibly the formation of 4-(dimethyl amino) benzophenone, according to previous studies [69].

**Figure 10:**
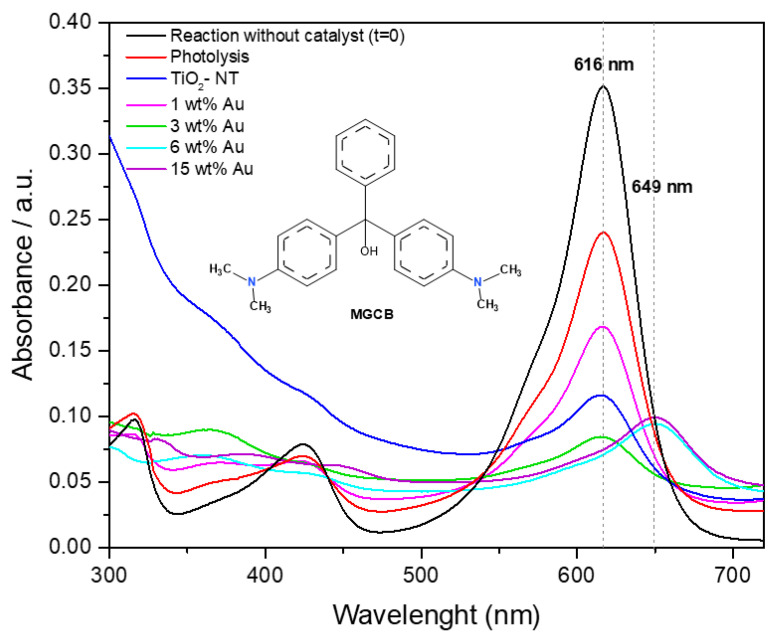
Comparison of UV–vis spectra of the decoloration reaction photocatalyzed with Au/TiO_2_-NT photocatalysts and MG dye solution as a reference.

It is worth mentioning that the increase in the metal load of the photocatalysts causes a bathochromic shift of the principal absorption band from 616 nm to 649 nm (6 and 15 wt% Au samples). This displacement can be observed in Figure 10 at a reaction time of 60 min. Additionally, the absorbance spectra of MG dye obtained at zero min of reaction and photolysis are presented. However, the change in the wavelength of the main absorption band of MG dye was observed during short reaction times (10 min). The red shift could be attributed to the presence of electron donor/acceptor groups on the aryl moieties, which are possibly promoted by the photocatalyst with high metal content because a higher gold nanoparticles density provides a greater number of surface available electrons affecting the extent of the red shift [70]. The inset of Figure 10 describes the structure of the malachite green carbinol base.

Derived from the UV–vis spectra for the decoloration of MG dye, we used the Lambert–Beer relation to determine the concentration and calculate the decoloration percentage through Equation (1).

(1) $$\%Decoloration=\frac{C_{0}-C}{C_{0}}x100$$

where *C*_0_ is the initial concentration of dye; *C* is the concentration at a time t.

**Figure 11:**
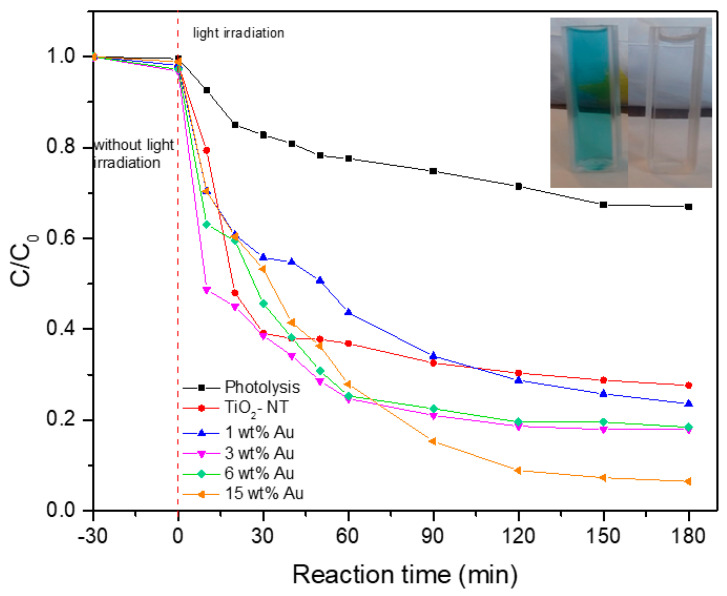
Decoloration of MG in the presence of pure TiO_2_-NT and Au/TiO_2_-NT photocatalysts.

The results of the application of the above equation are presented in Table 2. In addition, Figure 11 shows the variation in MG concentration as a function of irradiation time, as well as the effect on the dye concentration due to the catalyst without light irradiation, to establish the adsorption–desorption equilibrium of the catalysts. It is possible to observe that the capacity of the catalysts is not enough to achieve the green dye being removed by physical adsorption due to the low percentage of decoloration during the dark evaluation, and the better diminution of dye green is due to the photocatalytic process. It should be noted that the trend of decoloration reaction is highly favorable when the photocatalysts TiO_2_-NT and Au/TiO_2_-NT are used, as can be seen when compared to the reaction with photolysis. The inset in Figure 11 shows the MG dye solution before and after the decoloration reaction using a 15 wt% Au/TiO_2_-NT photocatalyst. It is well known that the dye photodecoloration increases with increasing catalyst amount and thus the number of active sites, which is the feature of heterogeneous photocatalysis [71,72]. However, the use of TiO_2_ nanotubes has an important effect on the decoloration of the dye, especially in the first 30 min of reaction, given that, as was previously documented, the photocatalytic activity is attributed to the functional groups present on its surface [73]. However, this capacity for decoloration is limited due to the low electron density compared to the metal-semiconductor coupling, especially in high metal load, where a higher electronic surface density allows a greater number of electrons available to continue the oxidation process and subsequent decoloration of the dye, such as the 15 wt% Au catalyst.

Although the photocatalysts with low Au load (3 and 6 wt%. Au photocatalyst, especially) show a significant decoloration capacity in the first minutes of the reaction, attributed to a convenient number of active sites and dispersion on the TiO_2_ nanotubes are not sufficient to continue the complete decoloration dye.

Table 2 presents the decoloration results obtained with the different photocatalysts, as well as band gap energy and main particle size determined from HAADF-STEM measurements.

The reaction rate constant k was determined considering that under the experimental conditions used, the photocatalytic curves follow pseudo-first order kinetic reaction, which is given by Equation (2).

(2) $$\ln\frac{C_{o}}{C}=Kt$$

where *C* is the reactant concentration, *K* is the reaction constant, and *t* is the reaction time. Kinetic parameters resulting from the application of this equation are presented in Table 2. In general, the Au/TiO_2_-NT materials are more efficient photocatalysts compared to the unmodified TiO_2_-NT, with higher decoloration percentages at 180 min and higher values of the rate constant. In the absence of gold nanoparticles, TiO_2_-NT decomposes MG dye with a rate constant of 54.76 *×* 10^−4^ min^−1^; this value is triplicated for 15 wt% Au photocatalyst. This increase in the total reaction rate related to the increase in Au content can be explained through the results of the characterization performed. Predominantly, the effect of metal nanoparticles in providing chemically active sites where relevant chemical transformations can take place with lower activation barriers than on the TiO_2_ semiconductor. In addition, Au nanoparticles can extend the lifetime of energy carriers reaching the surface of the semiconductor by increasing the electron-hole separation rates at the nanoparticle/semiconductor interface [34,66]. This, coupled with the fact that, as observed in the results obtained by UV–Vis spectroscopy of the photocatalysts, surface plasmonic resonance can contribute to the increase in the photodecoloration rate. One of the mechanisms proposed indicated that charge carriers are directly injected from excited plasmonic-metal nanostructures into the semiconductor surface. The strong interaction between Au nanoparticle and TiO_2_-NT semiconductor allows a fast of charge carriers. It means, in essence, that the metal nanoparticles act as a dye sensitizer, absorbing resonant photons and transferring the energetic electron formed in the process of the SPR excitation to the TiO_2_ semiconductor [63]. Another important issue is that the intensity of SPR and wavelength can be modulated by the composition, shape, and size of plasmonic nanoparticles [64,65].

**Figure 12:**
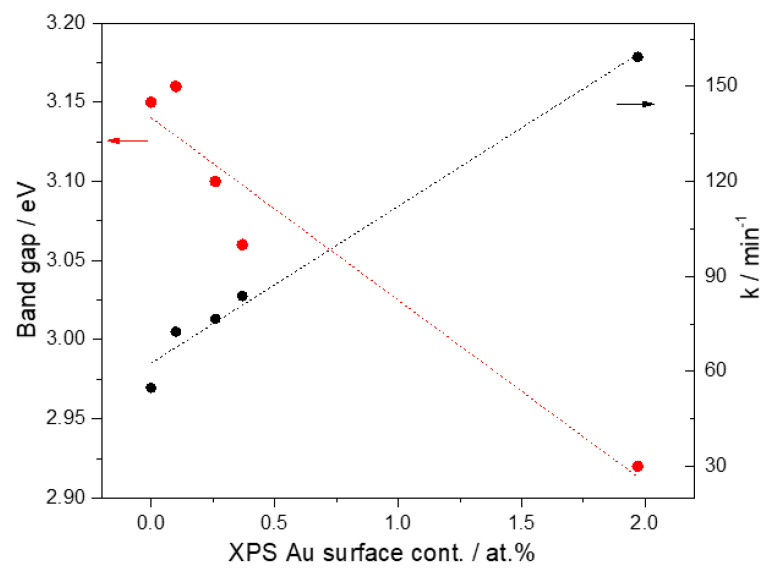
Linear dependence of semiconductor band gap (red arrow) and rate constant (black arrow) on the Au surface concentration determined by XPS.

In Figure 12, a linear correlation was observed between the photocatalytic rate constant and Au surface concentration determined by XPS. As mentioned before in our HAADF-STEM results, particle size remains constant even at the highest Au concentration; the higher Au nanoparticles, the higher electronic interaction between Au and TiO_2_ surface, which is revealed by a linear decrease in the semiconductor band gap, as can also be seen in Figure 12. 

## 5. Discussion

Various techniques for the incorporation of noble metal nanoparticles were described in several studies. Among the most common reduction methods, those using citrates [74,75] or sodium borohydride [76] as reducing agents allow obtaining monodisperse particles of uniform size. However, the size obtained is dependent on the subsequent heat treatment scheme, which can lead to particle growth or agglomeration, as well as higher energy costs. Various techniques for the incorporation of noble metal nanoparticles were described in several studies. Among the most common reduction methods, those using citrates or sodium borohydride as reducing agents allow obtaining monodisperse particles of uniform size. However, the size obtained is dependent on the subsequent heat treatment scheme, which can lead to particle growth or agglomeration, as well as higher energy costs. On the other hand, traditional synthesis techniques such as sol–gel and its variants lead to the same problem [77]. In order to overcome the drawbacks of the above-mentioned techniques, Au/TiO2 materials were prepared by VPI. The structural characterization reveals that nanoparticles are in a reduced state and in close relationship with the functional groups present in the support (Ti–O–Au), according to the X-ray diffraction and Raman spectroscopy analysis. Au nanoparticles present a high dispersion and a mean particle size around 3.0 nm for metal loading from 1 to 15 wt% and apparently have a semispherical form in a cuboctahedra geometry. The XPS results confirm that Au nanoparticles are in oxidation state Au^0^ even at high Au concentration. This means that during the VPI method used to prepare the photocatalysts, a reductive atmosphere is generated by the Au precursor decomposition, and highly dispersed reduced Au^0^ nanoparticles were obtained. For its part, Au nanoparticles present a surface plasmonic resonance evidenced by UV–Vis spectroscopy, which can improve the photocatalytic decoloration rate. According to previous studies, the photocatalytic efficiency of plasmonic metals is directly related to the intensity and wavelength of SPR; these parameters are dependent on the size, form, and composition of nanomaterials [34]. Nevertheless, the mechanism by which RPS can increase the concentration of charge carriers, and this, in turn, increase the photocatalytic activity, need to be studied. Likewise, photocatalytic activity correlates linearly with the surface Au concentration determined by XPS, which in turn decreases band gap energy of these photocatalysts due to an increment of the electronic interaction between Au nanoparticles and TiO_2_ semiconductor, yielding the electron-hole separation and more holes left by the electron migration contribute to enhance the photodecoloration efficiency. In this way, the high photocatalytic decoloration of malachite green dye using Au/TiO_2_-NT materials, especially 15 wt% Au sample, could be ascribed to a synergic effect between plasmonic Au nanoparticles and TiO_2−_ NT, which generate a high density of surface electrons that promotes the decoloration of the MG dye. 

## 6. Conclusions

The photocatalytic efficiency is related to four important parameters: (1) Metal load deposited on TiO_2_-NT. As noted above, the increased gold content boosts the number of surface-active sites available for catalysis. (2) Although Au particle size is a factor that directly impacts the surface area, the Au/TiO_2_-NT photocatalysts have a small particle size, and the increment in the metal load apparently does not affect the dispersion, and the particle size is maintained. This is directly related to the synthesis technique used to obtain the photocatalysts. (3) The electronic interaction between Au nanoparticles and TiO_2_ nanotubular, suggested by XPS and Raman results, must be related to the decrease in the band gap, which eventually results in an ion exchange process [49,67]. Furthermore, influence the photocatalytic performance of the systems under study by electron-hole separation. Thus, more holes left by the electron migration can facilitate the photodecoloration processes [29,33]. (4) The effect of SPR visualized by UV–Vis of Au nanoparticles on TiO_2_-NT improves the reaction rate allowing the decoloration of the MG dye. Thus, for the photocatalysts evaluated, the highest efficiency (93%) was obtained with a metal loading of 15%.

## Figures and Tables

**Table 1:** XPS parameters for Au/TiO_2_-NT photocatalysts.

Photocatalyst	Binding Energy (eV)	FWHM (eV)	Assignment	% at.
TiO_2_-NT	458.8 457.5	1.3 2.2	Ti^4+^ Ti^3+^	16.7 4.1
1 wt% Au/TiO_2_-NT	458.6 457.0 83.3	1.1 1.1 0.8	Ti^4+^ Ti^3+^ Au^0^	25.4 0.7 0.1
3 wt% Au/TiO_2_-NT	458.7 456.9 83.4	1.1 1.1 0.9	Ti^4+^ Ti^3+^ Au^0^	23.3 0.5 0.26
6 wt% Au/TiO_2_-NT	458.6 456.8 83.4	1.1 1.1 0.9	Ti^4+^ Ti^3+^ Au^0^	25.4 0.6 0.37
15 wt% Au/TiO_2_-NT	458.8 457.0 83.7	1.1 1.1 0.9	Ti^4+^ Ti^3+^ Au^0^	24.4 0.5 1.97

**Table 2:** Mean particle size, band gap energy, and kinetic parameters of the malachite green decoloration photocatalyzed by Au/TiO_2_-NT materials.

Catalysts	Mean Particle Size (nm)	Band Gap Energy (eV)	Rate Constant ^a^, *k* × 10^−4^ (min^−1^)	% Decoloration ^a^
Photolysis	--	--	17.55	31.19
TiO_2_-NT	--	3.15	54.76	72.15
1 wt%Au/TiO_2_-NT	3.1	3.16	72.54	75.98
3 wt% Au/TiO_2_-NT	3.1	3.10	76.54	81.51
6 wt% Au/TiO_2_-NT	2.9	3.06	83.77	81.05
15 wt% Au/TiO_2_-NT	3.2	2.92	159.30	93.47

^a^: At 180 min reaction time.

## Data Availability

Not applicable.
